# Foreign Correspondence

**Published:** 1855-11

**Authors:** N. E. Gage

**Affiliations:** Berlin


### *Foreign Correspondence of the N. H. Journal of Medicine.*

BERLIN, August 1, 1855.

MESSRS. EDITORS:—If there is any one thing of which the medical centres of Europe can boast preeminently, it is the means afforded for acquiring a knowledge of the sciences, which though not necessarily embraced in a medical course, ought at least to receive some attention from the student of our profession. There is no point in which our physicians are so remiss as this. It is rare that you find a medical man of any claims in Germany and indeed in Europe, who is not able to become the arbiter upon any ordinary scientific question which may arise in the course of his practice, and his intimate acquaintance with Latin, French and very often English, enables him to command the whole medical literature of the continent, and to learn of every new scientific discovery at the earliest day. You will find him also able to analyze the water of a well, or a farmers soil, to give evidence at an inquest upon toxicological points, to test the purity of drugs, and to take advantage of the medical botany of his country. I am sure that such qualifications are seldom found in America, and that the medical profession does not stand so high in public estimation in consequence. Were they professed by all, quackery would no more flourish there than here, and this country is certainly free enough from it. Here the profession is esteemed by all; the government is peculiarly favorable to it because it never meddles with politics, and the people are satified to leave their health to the care of men so learned in their profession and so assiduous in their studies.

When the term of student life with us is lengthened and the requirements for graduating doubled, our physicians will, as a mass, hold the same place in the community, and the necessity of studying in Europe be not so great as now. With the exception of the chair of comparative anatomy at Charleston, S. C., the Hersey Professorship at Cambridge, the Chandler, Lawrence and a few other scientific schools, I know of but few means, open to an American physician desiring to embellish his mind by a study of the collateral sciences.

I did not mean to preface this letter at such length, but the readers of the Journal will, I know, excuse an allusion to this important subject.

Among the collateral means of study here, the anatomical museum is first. It is in the university building, originally a palace. Though without a descriptive catalogue, its excellent arrangement makes up for this deficiency in great part. It is not so rich as the English Hunterian museum, yet one of the first in Europe. The specimens illustrating comparative Anatomy are very numerous, and the gigantic monsters of preadmite earth which fill one hall are among the most perfect yet discovered. A laboratory is attached to this department and lectures of Professors Remak, Peters, Ehrenberg and Muller and others, give the student a comprehensive view of the whole. The subject of human anatomy is also excellently represented; many of the injections being unusually fine. Some of the pathological specimens also are very valuable, those of hypetrophy and dropsies especially. I am almost tempted to mention a few of the best, but they must be seen to gain an idea of them. There is one skull which I remember as an example, more than a foot in diameter. It is that of a person who died of hydrocephalus. The surgical gallery is small but capitally arranged. There are divisions for disease of the bones and of joints, for fractures, luxations, clubbed-foot and hand, hernia, diseases of the sensitive organs, and cutaneous affections. The gem of this department is the collection of colored casts of syphilis. Every phase of this Protean disease is represented accurately. But what strikes the visitor to this collection particularly, is the vast number of monstrosities and lusus naturæ here collected. Here are double headed fœtusses, breasts joined like the Siamese twins, skeletons seven feet high, dwarfs, animals with three or four surplus legs, some with limbs growing upwards from the spinal column, birds with two heads and others having hoofs, others being a combination of bird and animal in the oddest proportions. It is an envious assemblage, though not without value, especially to the study of theoretical embriology. If any of our readers have seen Tenier's painting called the Temptation of St. Antony or a copy and have never visited this part of the Berlin Anatomical Museum, they can imagine the droll and whimsical specimens here contained.

The Natural History Museum is no less interesting. It comprises the Zoological collections at the University and in their Garden, the Royal Menagerie, the collection of minerals with the rich stones collected by Alexander Von Humboldt in Asia and South America, and the Botanical Garden. This last, with its thousands of exotics from all points of the earth, and its trees growing to the height of forty or fifty feet so far away from their natural soil, is rich indeed. Since the return of Mr. Lichtenstein, who was sent by the Prussian government, at the early part of this century, to reside in Southern Africa, and who brought back with him a vast collection of specimens pertaining to mineralogy, botany and zoology, the Berlin cabinets have no superior in Europe except the Jardines Plantes at Paris

The Medical department of the Royal and University Libraries here, are of great importance to the student. English books, however, are scarce, compared with those of other nations and the progress of the science is not shown in procuring the standards as they are published. The reading room has all of the European, and many American Medical publications, but is surrounded with so many restrictions as to be difficult of access. Private Medical reading rooms, however, give the student ample facilities in this respect. Those of your readers who may have heard the lectures of the late Professor of Obstetrics in the Boston school, since his European tour, will remember his allusion to the curious relics of our profession in the Cabinets here. I have found a few in the collection of metallic wares from the buried cities of Italy. Here are probes, directors with scoops, spatulas, lancets, scalpels, a a packed medicine case with little brass boxes instead of bottles, and forceps like the English polypus forceps, but whose object can hardly be guessed. The sharp teeth upon the blades show the instrument to have had a very powerful grasp, whatever its use. In the Egyptian Museum also, there is a medicine chest filled with alabaster vials, supposed to have belonged to a monarch. Besides this, the embalmed specimens of men and of sacred animals, a human monster without brains or spine embalmed, the brass hooks and knives of sharpened flint, by which the brains of the dead were extracted through the nostrils, are all curious to the student of our profession.

The facilities for dissection in Berlin are good. In the winter season the students pay particular attention to that study. Subjects though by no means so cheap as in Prague and some other places, can usually be obtained for about three dollars a piece. The government of Prussia is neither very favorable nor unfavorable to this study, and the chief obstacle which the student encounters, is the existence of benevolent societies whose funds are appropriated to procure a burial for friendless persons who die in the hospitals.

I find one feature in the relations of our profession and the government here, which is very superior. The inquests are all under the charge of medical men, and not in the hands of coroners, ignorant, as ours often are, and perfectly incapable of conducting an autopsy, or determining correctly the causes of death.

Besides the medical department of the Royal University, there is the military surgical school here, similar to the Imperial school in Vienna, the Military College of Dublin, and those in France. The young men who propose to join the medical staff of the army, and who pledge themselves to act as military surgeons for at least seven years, are here educated free, and have their ordinary expenses paid by government. As a class, these men are by no means brilliant students, and the effect of this gratutious instruction is much the same as in providing scolarships in literary institutions. In all the hospitals, however, through the country, the posts of house physicians are assistants and occupied by members of the military staff, who are detailed round to these places in turn. Thus the whole body is kept practically acquainted with surgical diseases, and ready to change their places for active service in the army, whenever an emergency demands.

It is singular that the cost of medicines, medical books, and instruments in this country is not two thirds as much as with us, and the fees for Medical attendance and surgery bear the same relation to ours. A medical man rarely becomes rich here ; unless he receives the patronage of noble families, or accomplishes some signal cures, a thousand dollars a year is a handsome income. The fee for ordinary professional visits is never more than fifty cents, even in the cities, except for the first visit which is seventy five, and for a night call which is double the usual price. The fee for consultation never exceeds two dollars, that for an ordinary midwifery case is from one and a half to four dollars, and never above seven.

The fees for surgical operations are as moderate. For cataract *never* above twelve dollars, extirpation of the eye the same, tracheotomy nine dollars, lithotomy thirty-five, amputation of thigh or humerus twelve, the reduction of dislocations four, the cæsarean section fifteen, for embryotomy seven, and for excision of the mamma twelve. These are the highest prices allowed; they are ordinarily about two-thirds, or one half as much. The fees are all established by law, as indeed is every thing connected with the profession. The term of study is fixed, and the qualifications for graduating. After graduating, a legal approval is necessary before one can practice, and this involves an examination ; as often as he changes his location, the hygiene of the country is attended to and enactments made upon this subject; the studies and practice of apothecaries are regulated and under legal inspection. In fine, every thing which with us falls to the duty of local societies to establish and local authorities to recommend is here regulated by general laws which are the same in city or country, all over the kingdom. Our legal profession is certainly not more under law, and yet the medical man is much better off for these restrictions. All his claims are limited to his own abilities as a well read physician. He has no fear of brethren going astray and practising irregular systems, and he can always retain the confidence of the community which knows that it is free from quacks or imposters. Among rich families, in the towns and cities, it is a very common thing to have the family physician call a moment twice a week to inquire after their health and to anticipate any disease. At the end of the year, they receive presents for this service, besides the regular fees for professional visits. It is an excellent custom, and serves to bind stronger and stronger the mutual friendship and confidence of physician and patient. In Prussia there is a minister of Religion, Education and Medical affairs. The central medical Bureau has a Director and five Judges. These are among the most eminent of our profession in the country. There is also a deputation from the medical body having a director and ten members. Besides this, each Province has its local court with its Judges, its Pharmaceutical Assessor, its Veterinary Assessor and other attaches. It is, in all, a splendid system and worthy of being followed in every country.

I found one custom of the middle ages, still existing here, which I had not expected. The barbers are yet to a certain degree barber surgeons. They are still called in to bleed a patient, to apply a leach or put on a cupping glass. I do not know but that they perform these offices well enough, but the mere idea of a physician leaving his patient to be bled a certain amount and taking no means to watch the effect of this abstraction, is ridiculous. The custom of connecting the surgical and shaving profession is not alone confined to Prussia. So late as the early part of this century, I forget the year, several English surgeons who had enlisted in the Swedish navy were dismissed from the service for refusing to *shave the crews.* You see nothing however of the striped pole here to indicate the white bandage and bloody wound as in England and with us. The symbol here is simply an oval plate of brass, a primitive shaving dish I imagine from its looks.

I find I shall hardly have room in this letter to speak of the diseases of the climate and a few other topics. I must leave these to another time. I trust that the remarks which I have made in regard to the condition of our profession here in Germany and the advantages for studying the collateral sciences, will have a value and interest to the readers of the Journal.

N. E. GAGE.

